# Professional agency in the classroom and burnout among early career teachers in China

**DOI:** 10.3389/fpsyg.2024.1412446

**Published:** 2024-11-06

**Authors:** Liyuan E, Auli Toom, Janne Pietarinen, Tiina Soini, Kaisa Haverinen, Kirsi Pyhältö

**Affiliations:** ^1^Centre for University Teaching and Learning, Faculty of Educational Sciences, University of Helsinki, Helsinki, Finland; ^2^School of Applied Educational Science and Teacher Education, University of Eastern Finland, Joensuu, Finland; ^3^Faculty of Education and Culture, Tampere University, Tampere, Finland; ^4^Centre for Higher and Adult Education, Faculty of Education, Stellenbosch University, Stellenbosch, South Africa

**Keywords:** teacher learning, professional agency, teacher burnout, structural equation modeling, Chinese context

## Abstract

The aim of the present study is to investigate early career teachers’ professional agency in the classroom. In addition, the association between early career teachers’ professional agency in the classroom and the burnout they experienced was examined. In this study, 779 early career teachers, teaching in primary and junior secondary schools in China, responded to the study survey in 2021. The Mplus statistical package (version 8.4) was used to conduct the analysis and the data were analyzed using structural equation modeling. The results indicated that early career teachers’ professional agency in the classroom consists of three elements: motivation, self-efficacy beliefs, and skills to manage new learning, which entails building a collaborative environment by transforming teaching practices and reflection in the classroom. The results also showed that early career teachers’ professional agency in the classroom was negatively related to their burnout.

## 1 Introduction

It has been suggested that teachers’ professional agency plays an important role in enhancing student learning, facilitating professional development, and implementing educational innovations (Borko, 2004; Lieberman and Pointer Mace, 2008). Professional agency refers to the intentional management of learning calling for teachers’ motivation and efficacy beliefs and intentional acts to manage learning (Sachs, 2000; Soini et al., 2016). Teachers’ professional agency is always socio-culturally embedded and thus may vary across the context (Edwards, 2015). However, the most of the empirical research on teachers’ professional agency has been conducted in Western contexts, for example in North America (e.g., Fu and Clarke, 2017), the Netherlands (e.g., Van der Heijden et al., 2015), the UK (e.g., Edwards, 2005), and in Finland (e.g., Eteläpelto et al., 2015; Pyhältö et al., 2015). This indicates a need for more studies to explore teachers’ professional agency in other regions such as Asia, especially using a quantitative method design (Cong-Lem, 2021).

Teacher burnout entailing teachers’ experience of inadequacy, exhaustion, and cynicism, has been considered to be a serious occupational problem in the school setting (Pietarinen et al., 2013a; Shen et al., 2015). It could cause undesirable outcomes such as lower levels of job commitment and satisfaction, worse physical and mental health, and teacher attrition (Hakanen et al., 2006; Heikonen et al., 2017; McLean et al., 2019; Skaalvik and Skaalvik, 2017; Vanderslice, 2010). To alleviate the negative consequences of burnout, it is important to understand its antecedents. Some studies have suggested that teachers’ professional agency, including motivation, self-efficacy, and strategies, may protect them from burnout (Dicke et al., 2014; Fernet et al., 2012; Oliveira et al., 2021). Nevertheless, we know little about the interrelation between teachers’ professional agency and burnout. Thus, the aim of this study is to bridge the gap in the literature by exploring early career teachers’ professional agency in the classroom and its association with burnout in China. Early career teachers in this study are defined as those who are in their first 5 years of teaching (Lavigne, 2014). The reason for focusing on early career teachers is that the first few years of teaching are important for teachers’ professional growth and commitment to their teaching career, and are regarded as having long-term impacts on their teaching effectiveness, job satisfaction, and career length (Hulme and Wood, 2022; McCormack et al., 2006). Also, gender as a demographic predictor for teacher burnout and the mediator role of professional agency will be examined. This study mainly contributes to enriching the understanding of how Chinese early career teachers perceive their professional agency in the classroom and how their professional agency in the classroom is related to burnout.

### 1.1 Early career teachers’ professional agency in the classroom

Teachers’ agentic role has been expected in the professional workplace (Edwards, 2015; Ketelaar et al., 2014). Research provided several perspectives on the concept of teacher agency as related to individual behavior or social system (Eteläpelto et al., 2013). In line with recent studies that understand professional agency as an interdependence between individual and social context (e.g., Imants and Van der Wal, 2020), this study considers teachers’ professional agency to be their capacity to manage new learning intentionally and responsively (Pyhältö et al., 2015; Soini et al., 2016). In this approach, learning is the object of professional agency (Edwards, 2005). However, becoming an agent in learning entails not only teachers’ motivation and self-efficacy but also intentional acts to facilitate learning (Edwards, 2005; Sachs, 2000; Turnbull, 2005; Van Eekelen et al., 2006). Thus, teachers’ professional agency is an integrative concept including the interrelated elements of motivational and attitudinal resources and abilities to promote new learning (Pyhältö et al., 2015; Vermunt and Endedijk, 2011). Teachers’ professional agency is highly relational and is influenced and constructed by various contexts and social interactions (Edwards, 2005). Therefore, it is not a stable trait, but it changes over time, depending on the interactions with pupils and contexts (Priestley et al., 2015).

The classroom and professional community form the primary contexts of teachers’ work in which they enact their professional agency. In this article, the specific context that will be explored is classroom. In the classroom, teachers learn by experimenting with innovative teaching methods and building collaborative environments and reflecting on their teaching practices (de Vries et al., 2013; Hoekstra et al., 2009). Thus, the elements of teachers’ professional agency (i.e., motivation, self-efficacy and intentional activities) are ingrained in two modes: collaborative environment and transformative practice, and reflection in classroom (Soini et al., 2016).

Learning by creating a collaborative learning environment and engaging in transformative practice entails the flexible and responsive adaptation of classroom activities to promote reciprocal learning (Järvelä et al., 2010; Soini et al., 2016). This calls for developing interpersonal skills and dynamic coordination of the learning environment with pupils (Sachs, 2000). Kwakman (2003) found that teachers with the capacity to facilitate a collaborative environment can perceive others (i.e., pupils) as being crucial resources for their learning. Moreover, they are more likely to adjust the classroom atmosphere to meet the needs of different groups of pupils and create a functional relationship with them (Darling-Hammond, 2008; Martin and Dowson, 2009). Early career teachers have been found to experience problems in using pupils as a resource to modify teaching practices, in meeting their learning needs, and in constructing reciprocal learning environments (Edwards, 2005; Zambrano et al., 2019). However, early career teachers have the chance and capacity to develop pedagogical practices as well as facilitate teacher-student interactions to improve their ability for collaborative learning in the classroom (Alhija and Fresko, 2010; Eteläpelto et al., 2015).

Learning through reflection involves active observation, monitoring, and meaning making to improve teaching and enhance learning in the classroom (Naidoo and Kirch, 2016). Several studies have shown that reflection is an important contributor to teachers’ active learning (Meirink et al., 2009). For example, Husu et al. (2008) found that early career teachers’ ability to reflect builds a foundation for their professional agency, and hence their professional agency development is based on considering pedagogical activities from a reflective perspective (Tilson et al., 2017). Becoming reflective practitioners allows early career teachers to observe and adopt observed instructional practices in teaching and recognise the interaction between knowledge, beliefs, strategies, contexts, and identity (Korthagen et al., 2006). These activities can guide teachers’ actions in the interactions between teachers and students, and therefore support collaborative learning in the classroom (Claessens et al., 2016).

Compared to Western culture, China presents a more collectivist culture (Zhu and Leung, 2011). McInerney et al. (2008) have suggested that self-regulative behaviours may involve obedience, working for pre-set aims decided by others, diligence, respect for direct teaching, memorisation, and rote learning within collectivist cultures. Thus, Chinese teachers might be more likely to adjust personal demands to others and constrain personal desires to be receptive to others (Cheng and Lam, 2013). There is tentative evidence that Chinese teacher learning may come from both intrinsic and extrinsic motivation and the combination of these two kinds of motivation might contribute to better results (Zhu and Leung, 2011).

China has undergone education and teacher education reform since the late 1990s (Zhou, 2014), and teachers have been expected to become curriculum developers, active implementers of innovation, and facilitators of pupil learning (Lee et al., 2013). On the other hand, the reform has added responsibilities and accountabilities to teachers for student learning outcomes and well-being (Zeng and Day, 2019). Some researchers have suggested that a performative culture can diminish teacher agency (Priestley et al., 2015). However, teachers were able to enact strong agency when they were confident in their capacity in the face of performativity (Helsby, 1999). These findings provide the primary background for investigating early career teachers’ professional agency in China for this study.

### 1.2 Early career teachers’ burnout, gender, and professional agency in the classroom

Burnout can be conceived of as a process (Schwarzer et al., 2000), which develops over a prolonged period of stressful encounters (Skaalvik and Skaalvik, 2010). Burnout was first introduced by Freudenberger (1974) emphasized emotional exhaustion as the consequence of long-term overwork and overextension. Maslach and Jackson (1981) extended this initial definition of burnout to include three distinctive symptoms: inadequacy, exhaustion, and cynicism (Hakanen et al., 2006; Maslach et al., 2001). For teachers who work in the schools’ social context, inadequacy means feeling insufficiency in professional competence, especially in teacher-student interaction; exhaustion refers to basic stress response, lack of emotional and cognitive energy, and feeling tired in the workplace; and cynicism is characterized by indifference to work, a distant attitude toward pupils, parents, or colleagues, and low organizational commitment (Hakanen et al., 2006; Maslach et al., 2001; Pietarinen et al., 2013b).

Teachers are in a profession that has been reported to experience the highest risk of burnout (Hakanen et al., 2006; Skaalvik and Skaalvik, 2017). Teacher burnout is a global concern (Aloe et al., 2014), and China is also included in this rising phenomenon (Cheng et al., 2023). Chinese teachers usually face stressful work conditions with large class sizes and relatively low rewards (Wang et al., 2015). Also, the emphasis on student academic outcomes and competitive examination seems to be another stressor (Zhang and Zhu, 2007). As a result, teachers in China are possibly more likely to be vulnerable to burnout. Research has provided some insights regarding the factors that predict burnout.

Gender has been related to teacher burnout (e.g., Gupta and Rani, 2017) and elements of teachers’ professional agency, i.e., self-efficacy for learning (Klassen and Chiu, 2010). The findings on burnout regarding gender are inconsistent. Some researchers found that female teachers are at a higher risk of experiencing exhaustion than male teachers (Antoniou et al., 2006), but others have found that they are at a lower risk of experiencing exhaustion than male teachers (Anderson and Iwanicki, 1984; Sak, 2018). Comparisons of professional inadequacy levels differed in that female teachers have experienced higher rates (Lau et al., 2005) and lower rates (Saloviita and Pakarinen, 2021), compared to male teachers. Regarding cynicism, male teachers have scored higher than their female counterparts (Lau et al., 2005). Regarding gender and the elements of professional agency, findings for the association are also mixed. For example, Cousins et al. (1996) found that male teachers had a higher self-efficacy, while female teachers have been found to have higher self-efficacy and higher motivation in other studies (Fives and Looney, 2009; Handayani, 2016). Additionally, based on the association of gender with teacher burnout and professional agency, professional agency may play a role in mediating the relationship between gender and teacher burnout.

Active learning allows teachers to modify their learning environment and develop their abilities (Cooper et al., 2020; Hoekstra et al., 2009). The impact of teachers’ professional agency, namely teachers’ motivation to learn, self-efficacy for learning, and intentional behaviours to facilitate learning, has been recognized in buffering burnout symptoms (Hakanen et al., 2006; Oliveira et al., 2021; Ryan and Deci, 2016; Schwarzer and Hallum, 2008; Yli-Pietilä et al., 2023; Zeng et al., 2019). Early career teachers experienced more burnout than experienced teachers (Gavish and Friedman, 2010). It has been shown that early career teachers not only have to teach, but they have to also learn to teach (Feiman-Nemser, 2001), and therefore they are more likely to face new challenges like classroom management issues (Gholam, 2018; Veenman, 1984). Despite the difficulties in the initial years of teaching, their interpretation of these challenges influences their well-being (de Vries et al., 2013). This means that teachers could perceive difficulties as being learning opportunities to direct and re-direct their learning (Pyhältö et al., 2015). These efforts may enable teachers to reduce the risk of burnout.

## 2 Aim of the study

**Figure 1:**
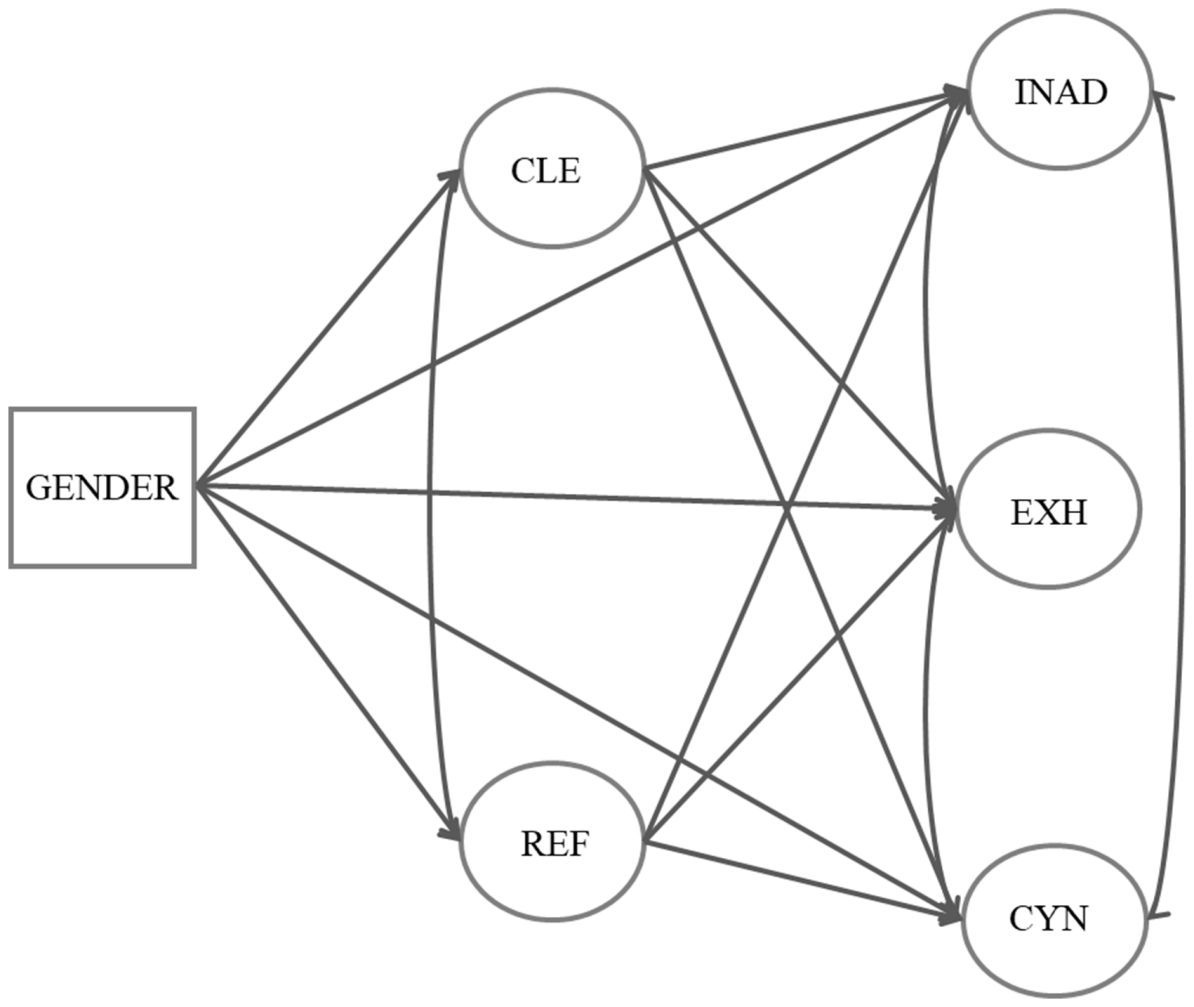
Hypothesized model of gender, the components of teachers’ professional agency in the classroom (CLE and REF) and teacher burnout indicators (INAD, EXH, and CYN). CLE = Collaborative environment and transformative practice; REF = Reflection in classroom; INAD = Inadequacy; EXH = Exhaustion; CYN = Cynicism.

This study aims to gain a better understanding of early career teachers’ perceptions of professional agency in the classroom in China. In addition, the interrelation between their professional agency, burnout, and gender was examined. Based on earlier research, the following hypotheses were tested (see Figure 1):

*H1:* Early career teachers’ sense of professional agency in the classroom consists of interrelated components including collaborative environment and transformative practice and reflection in classroom (Edwards, 2005; Pyhältö et al., 2015; Sachs, 2000).

*H2:* Early career teachers’ sense of professional agency in the classroom is negatively related to their burnout: inadequacy, exhaustion, and cynicism (Aloe et al., 2014; Skaalvik and Skaalvik, 2007; Soini et al., 2016).

*H3:* Gender predicts early career teacher burnout, and this effect is mediated by their professional agency in the classroom (Antoniou et al., 2006; Fives and Looney, 2009; Klassen and Chiu, 2010).

## 3 Methods

### 3.1 Research context

Chinese compulsory education includes primary school and junior secondary school. Primary school teachers are typically responsible for teaching grades 1 to 6 (pupils aged 6–11 years). Junior secondary school teachers typically teach grades 7 to 9 (pupils aged 12–14 years). The nine years of compulsory education are funded by the government. Teacher candidates are required to pass a national teacher qualification examination including a paper-and-pen exam on rudimentary content knowledge and pedagogical knowledge and an interview mainly on teaching competence to obtain a teaching certificate to teach in schools (Ye et al., 2019). All Chinese early career teachers are required to participate in comprehensive mentoring programmes (OECD, 2015). One-to-one mentoring is a significant way to develop early career teachers’ capacity in the first one to three years [Chen, 2006, as cited in Lu et al., 2020].

### 3.2 Participants

The present study included primary and junior secondary school early career teachers with a maximum of 5 years of teaching experience in China. The data used in this research were collected in spring 2021 (*N* = 779). A non-probability sampling method was applied. The participants were reached with the assistance of teachers, researchers, and teacher educators who work in different universities and schools in China to answer or disseminate the online survey. The sample included 19 provinces and four municipalities. Most of the sample was from Jilin province (*N* = 632, 81.1%). The participants included primary school teachers (*N* = 431; 55.3%) and junior secondary school teachers (*N* = 348; 44.7%). The respondents’ mean age was 27.6 years (SD = 3.57; min/max: 20/40 years), with the majority being women (*N* = 674; 86.5%) and the minority being men (*N* = 105; 13.5%). Of these participants, 56.9% taught in rural areas (*N* = 443), and 43.1% taught in urban areas (*N* = 336). The mean teaching experience was 1.75 years (SD = 1.41; min/max = 0/5 years). Among the respondents, 56 had associate bachelor’s degrees (7.2%), 604 held bachelor’s degrees (77.5%) and 119 possessed graduate degrees (15.3%). The participation of all the respondents was voluntary, and the participants were informed about the study before the data collection.

### 3.3 Measures

The data for the present study were collected with a professional agency survey (Pietarinen et al., 2013a; Soini et al., 2016). The two scales measuring (a) professional agency (10 items) and (b) socio-contextual burnout (9 items) were selected for this study. The professional agency scale (Pyhältö et al., 2015; Soini et al., 2016) measures two factors of early career teachers’ professional agency in the classroom: collaborative environment and transformative practice (6 items); and reflection in classroom (4 items). As far as we know, there are few well-researched diagnostic scales for measuring teachers’ professional agency. The professional agency in the classroom scale was designed to measure the key components teachers’ professional agency in the classroom and has been validated in earlier studies (e.g., Soini et al., 2016). The socio-contextual burnout scale draws on Maslach and Jackson’s (1981) burnout scale and Elo et al.’s (2003) single-item stress scale for measuring teachers’ perceived exhaustion. The socio-contextual burnout scale measures three factors of early career teacher burnout in line with the conceptual definition: inadequacy in teacher-pupil interaction (3 items), exhaustion (3 items), and cynicism towards the teacher community (3 items) (Pietarinen et al., 2013a). The validity and reliability of the socio-contextual burnout scale have been examined and supported in previous studies (e.g., Pietarinen et al., 2013a; Yli-Pietilä et al., 2023).

The original scales and items for this study were in English. Therefore, we utilized Brislin’s (1970) back-translation procedures to ensure the quality of translation and adaption to the Chinese context. First, two bilingual researchers translated the English version scales into Chinese independently. The translated instruments of each researcher were evaluated by another researcher. The differences between these two translation versions were discussed between them until reaching an agreement. Then, the Chinese version of the scales was back-translated into English by the third bilingual researcher. Finally, the original English version and back-translated version were compared and discussed with research team members who have developed the scales to identify the differences regarding some concepts and guarantee the similar meaning of the translated items.

The scales, items, and Cronbach’s alphas are shown in Table 1.

**Table 1:** The scales and items for exploring early career teachers’ sense of professional agency in the classroom and burnout.

Scales*			Cronbach *α*
**(1) Teacher’s professional agency in the classroom**			
Collaborative environment and transformative practice (CLE)			0.90
Cle11: I’ve been able to build functioning interactive relationship with my pupils			
Cle12: I’m able to create a nice atmosphere together with my students			
Cle13: When planning my work, I’m able to utilize the feedback I get from my pupils			
Cle14: I can modify my teaching to adjust to different group of pupils			
Cle15: I’m able to find teaching methods to engage even the most challenging group of pupils			
Cle16: I’m able to find ways to support the learning processes of all my pupils			
Reflection in classroom (REF)			0.86
Ref21: I still want to learn a lot about teaching			
Ref22: I’d like to understand young people’s ways of thinking and acting better			
Ref23: I regularly endeavor to estimate my success in teaching situations			
Ref24: I think we can all learn something in a teaching situation			
**(2) Socio-contextual teacher burnout**			
Inadequacy (INAD)			0.87
Inad11: The challenging pupils make me question my abilities as a teacher			
Inad12: I often feel I have failed in my work with pupils			
Inad13: Dealing with problem situations considering my pupils often upsets me			
Exhaustion (EXH)			0.76
Exh21: Stress means a situation in which a person feels tense, restless, nervous or anxious or is unable to sleep at night because his/her mind is troubled all the time. Do you feel this kind of work-related stress?			
Exh22: I feel burnt out.			
Exh23: With this work pace I do not think I’ll make it to the retiring age			
Cynicism (CYN)			0.89
Cyn31: I’m disappointed in our teacher community’s ways of handling our shared affairs.			
Cyn32: In spite of several efforts to develop the working habits of our teacher community they have not really changed			
Cyn33: I often feel like an outsider in my work community			

*The item scale: completely disagree 1 2 3 4 5 6 and 7 completely agree. For single stress item (Exh21) the scale is from one to ten: Not at all 1 2 3 4 5 6 7 8 9 and 10 very much.

All the items were rated on a 7-point Likert scale, ranging from 1 (completely disagree) to 7 (completely agree) (excluding the stress item, which was rated on a 10-point Likert scale), as the Likert scales applied in the original instruments (e.g., Pietarinen et al., 2013b; Soini et al., 2016; Elo et al., 2003; Maslach and Jackson, 1981). The percentage of missing values per item was 0. The measurement invariance in terms of professional agency and burnout scales between the two gender groups, male (*n* = 105) and female (*n* = 674), was tested with the configural, metric, and scalar models. The models for configural, metric, and scalar invariance were compared by examining changes in Root Mean Square Error of Approximation (RMSEA), Comparative fit index (CFI), and Tucker-Lewin Index (TLI) (Chen, 2007). Models for metric and scalar invariance were supported for the professional agency and burnout scales across the two participant cohorts.

### 3.4 Data analyses

**Figure 2:**
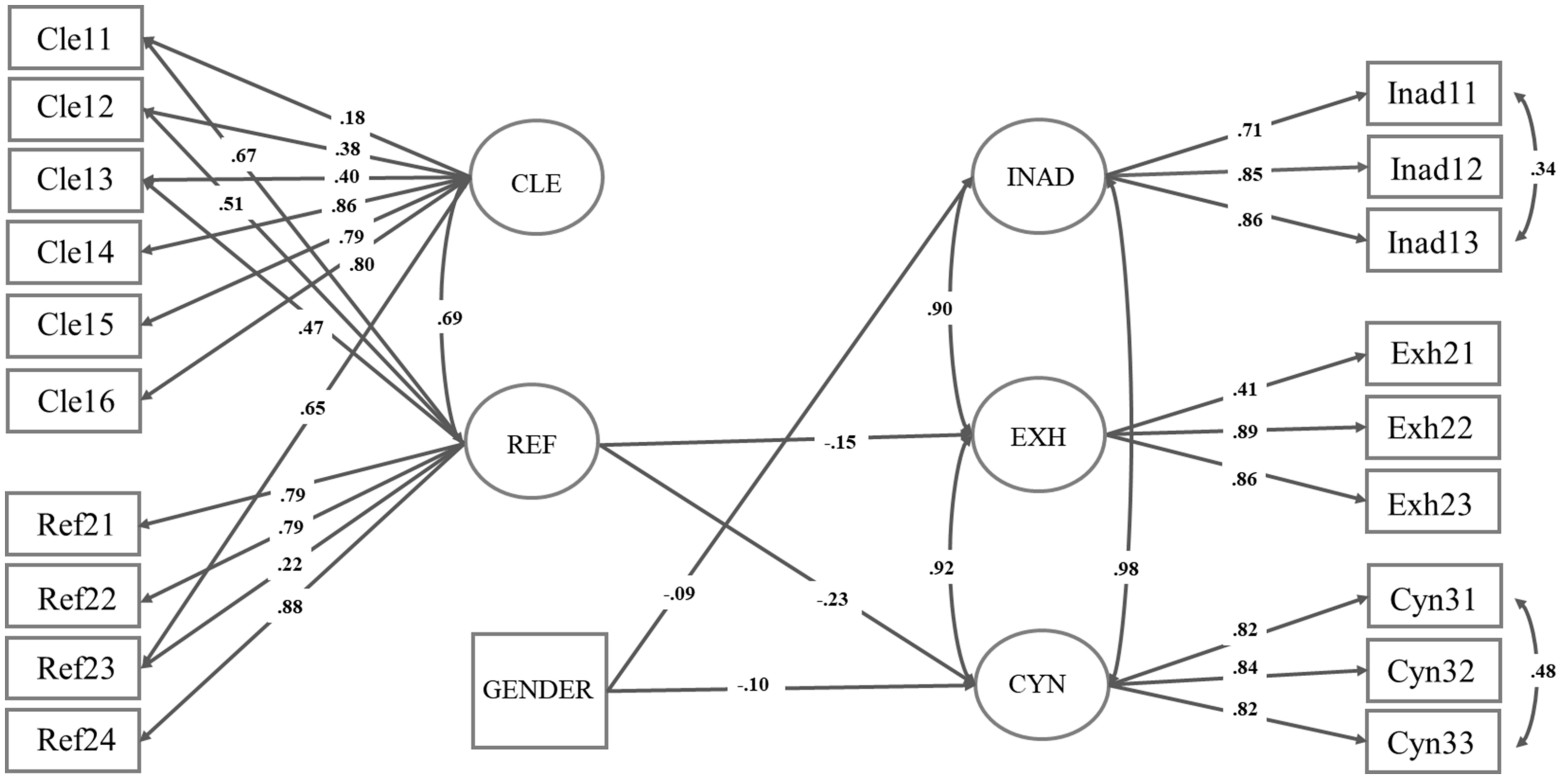
Standardised model: *χ*^2^ (136, *N* = 779): 565.99, *p* < 0.001; RMSEA = 0.06 (90% C.I. = 0.06–0.07); CFI/TLI = 0.95/0.93; SRMR = 0.04; *p* level < 0.001. CLE = Collaborative environment and transformative practice; REF = Reflection in classroom; INAD = Inadequacy; EXH = Exhaustion; CYN = Cynicism.

Firstly, Cronbach’s alphas of the scales were examined for reliability analyses. Secondly, the confirmatory factor analysis allowed for the analysis of the relations between variables and latent factors, and therefore statistically decide the extent of consistency of the hypothesized model with the data (Muthén and Muthén, 1998–2015). Finally, the hypothezised model was tested using structural equation modeling. The data analysis was performed by using the Mplus statistical package (Muthén and Muthén, 1998–2015). The model parameters were estimated with an MLR procedure, which produces maximum likelihood estimates with standard errors and *χ*^2^ test statistics that are reasonable for non-normality (Muthén and Muthén, 1998–2015). The goodness-of-fit of the estimated standardised model was evaluated by using the χ^2^ test, Comparative fit index (CFI), Tucker-Lewin Index (TLI), Root Mean Square Error of Approximation (RMSEA), and Standardised Root Mean Square Error of Approximation (SRMR). The goodness-of-fit of the hypothezised model [*χ*^2^ (136): 565.99, *p* < 0.001; CFI = 0.95; TLI = 0.93; RMSEA = 0.06; SRMR = 0.04] indicated a good fit (Hu and Bentler, 1999; Muthén and Muthén, 1998–2015). The model was specified by adding cross-loading items and residual covariances (see Figure 2) that were acceptable with respect to the theoretical assumptions (Soini et al., 2016).

## 4 Results

### 4.1 Components of early career teachers’ sense of professional agency in the classroom and burnout

The aims of the study were to determine the key elements of early career teachers’ sense of professional agency in the classroom and investigate the interrelation between their professional agency in the classroom and burnout. The results showed that the bivariate correlations between the components of professional agency in the classroom and burnout were statistically significant. The sample means, standard deviation, minimum and maximum values, and correlations for all factors are presented in Table 2.

**Table 2:** Means, standard deviations, minimum and maximum, and correlations between all factors of the scales (Observed means were used in scale correlations at *p* level < 0.05).

	1.	2.	3.	4.	5.
1. CLE	–				
2. REF	0.82	–			
3. INAD	−0.12	−0.09	–		
4. EXH	−0.09	−0.12	0.70	–	
5. CYN	−0.08	−0.15	0.82	0.72	–
*M*	5.60	6.02	3.83	3.78	3.30
SD	1.02	1.02	1.79	1.72	1.89
Min	1.00	1.00	1.00	1.00	1.00
Max	7.00	7.00	7.00	7.00	7.00
*α*	0.90	0.86	0.87	0.76	0.89

CLE = Collaborative environment and transformative practice; REF = Reflection in classroom; INAD = Inadequacy; EXH = Exhaustion; CYN = Cynicism. The stress item was rescaled from a 10-point Likert scale to a 7-point Likert scale using linear transformation for the descriptive statistics.

Early career teachers in China perceived their ability to learn in the classroom as being quite high in terms of the components of professional agency [Mean^(min-max)^ = 5.60–6.02]. They perceived the importance of building a reciprocal learning environment and learning from continuous reflection on their teaching practices (CLE, *M* = 5.60; REF, *M* = 6.02). Furthermore, the results indicated that early career teachers perceived their burnout at a moderate level [Mean^(min-max)^ = 3.30–3.83]. More specifically, early career teachers were not feeling high levels of professional inadequacy in teacher-student interaction, exhaustion, or indifference towards work (INAD, *M* = 3.83; EXH, *M* = 3.78; CYN, *M* = 3.30).

### 4.2 Interrelations between early career teachers’ sense of professional agency in the classroom and burnout

As shown in Figure 2, the factorial structure partly confirmed the first hypothesis (H1), that early career teachers’ sense of professional agency in the classroom was composed of the motivation to learn, self-efficacy beliefs, and intentional behaviours to facilitate learning. The results revealed that both latent factors, constructing a collaborative environment and transformative practice (CLE, 6 items, *α* = 0.90) and reflection in classroom (REF, 4 items, *α* = 0.86), were key and positively correlated elements for early career teachers’ professional agency (*r* = 0.69).

The model also showed that the elements of early career teachers’ professional agency was related to their burnout (H2). However, the impacts of the elements of early career teachers’ sense of professional agency on their experienced inadequacy, exhaustion, and cynicism differed from each other (see Figure 2). Specifically, a statistically significant negative relationship (−0.15) was found between early career teachers’ reported reflection in the classroom and the exhaustion (*R*^2^ = 0.03). Moreover, their perceived capacity for critical reflection (−0.23) statistically significantly reduced cynicism (*R*^2^ = 0.03), although the *R*^2^ represented a small percentage of variation. However, early career teachers’ reported capacity to reflect on their classroom interactions and practices was not associated with their perceived inadequacy in teacher-student interactions. Furthermore, early career teachers’ reported ability to construct a collaborative environment was partly connected to their perceived capacity to reflect on teaching (i.e., see the observed cross-loading items in Figure 2), and hence, it was not effective in reducing directly their burnout. This indicates that the two modes of early career teachers’ professional agency (i.e., collaborative learning and transformative practice and reflection in classroom) function partially through each other, but these two modes of professional agency buffer burnout differently.

### 4.3 Effects of gender on early career teachers’ sense of professional agency and burnout

The third hypothesis (H3) was that gender influences early career teacher burnout and this effect is mediated by their professional agency. The results showed that gender had a direct effect on burnout. More specifically, female early career teachers were more likely to experience inadequacy (*p* < 0.05) and cynicism (*p* < 0.01) compared to male early career teachers, although the effect of gender on exhaustion is not significant. Moreover, the results revealed that gender did not have a significant indirect effect on early career teacher burnout through their professional agency.

## 5 Discussion

### 5.1 Methodological reflection

The construct validity of the scales used in this study was acceptable and previously tested (see also Pietarinen et al., 2013b; Soini et al., 2016). The scales were first applied in China and showed an adequate fit to the data in this study although with cross-loadings and residual covariances. Thus, further construct validation of the scales and extra caution when interpreting the results are needed. Moreover, utilising a self-reported survey might be not enough to capture the complexity of Chinese teachers’ professional agency in the classroom, although it is the most common approach to typically investigating personal thoughts, feelings, or behaviours from a large number of participants (Duckworth and Yeager, 2015). It would be beneficial to explore the context-embedded nature of professional agency and its association with burnout among teachers in the China by using interviews and observations which may further help to develop the scales in the future. Also, the differences within demographic predictors (i.e., degree level) in teachers’ professional agency and burnout could be further examined. Finally, the study sample may be biased since most of the participants were from Jilin province (81.1%).

### 5.2 Findings in the light of the previous literature

The results confirmed our hypothesis that building a collaborative environment by adapting pedagogical practices and enacting reflection deliberately comprises core components of teachers’ professional agency in the classroom. This means that both elements, including building a collaborative learning environment by responsively adapting pedagogical practices and engaging in continuous reflection in the classroom, are required to construct early career teachers’ sense of professional agency in the classroom. However, these two components of early career teachers’ sense of professional agency have cross-loadings. Early career teachers’ reported capacity to regularly estimate their success in teaching situations cross-loaded on collaborative environment and transformative practice. The reason for this might be that the notion of success may involve significant others (i.e., pupils) embedded in the collectivist culture of China (Watkins, 2000). At the same time, early career teachers’ perceived ability to create and maintain a nice atmosphere and functional teacher-student relationship as well as to use pupils as a learning resource to plan their work cross-loaded on reflection in the classroom. A plausible reason might be that the teacher-student relationship tends to be hierarchical in China, which means teachers tend to dominate the process of education, and students are more likely to conform to the guidance provided by teachers (Li and Du, 2013). The results expand our understanding of the situational characteristics of teachers’ professional agency in China.

Moreover, the results suggest that early career teachers’ sense of professional agency seems to be negatively related to their burnout. This means that early career teachers perceived capacity to actively manage new learning in the classroom might reduce their risk of burnout. However, the relationship between early career teachers’ sense of professional agency and burnout is complicated. Specifically, early career teachers’ ability to reflect critically in the classroom can reduce two components of burnout including exhaustion and cynicism, although the effect size seemed relatively low. The results are in line with some prior studies showing that English as a foreign language teachers’ reflection is negatively related to their burnout (Shirazizadeh and Moradkhani, 2018). The reason for the small effect size might be that we focused on investigating the association between teachers’ professional agency in the classroom and burnout, but burnout may also be related to professional agency in the professional community. Especially in the context of China, teaching is a kind of collective activity (Paine and Ma, 1993), which means Chinese teachers are used to working together, observing each other, and sharing resources and ideas (Tan, 2013). Moreover, the social nature of inadequacy and cynicism might be more likely to be reduced by active participation in learning within professional community (e.g., Van Droogenbroeck et al., 2014). Therefore, teachers’ professional agency in the professional community might play a more important role in regulating burnout. However, early career teachers’ reflection does not reduce their inadequacy in teacher-student interactions. The results are in line with previous research, which found that inadequacy develops separately compared to exhaustion and cynicism (Lee and Ashforth, 1993). These imply that early career teachers seem to reflect their learning, which buffers their exhaustion and cynicism simultaneously. Therefore, early career teachers’ perceived capacity to reflect is not purely internal processing of pupils’ feedback or estimating their own success in teaching situations, but is also socially shared professional processing that seems to buffer alienation from the professional community’s development work.

Surprisingly, our results suggest that the three components (inadequacy, exhaustion, and cynicism) of early career teacher burnout are not directly related to their ability to create a collaborative learning environment. This partly contradicts some previous studies that have found the importance of building a functional teacher-student relationship for teachers’ burnout (Heikonen et al., 2017; Soini et al., 2016; Spilt et al., 2011). The reason might be that early career teachers’ reported capacity in constructing a collaborative environment was partly related to their perceived ability to reflect on pedagogical practices (i.e., see the observed cross-loading items in Figure 2).

In line with the findings of some previous studies, our results showed that female early career teachers were more likely to experience professional inadequacy compared to male early career teachers (Burke and Greenglass, 1989; Lau et al., 2005). Moreover, our study found that cynicism tended to be higher for female early career teachers than for male early career teachers. This contradicts prior studies by suggesting that male early career teachers generally showed more cynicism than females (Lau et al., 2005; Schwab et al., 1986). It is possibly because several aspects of collaboration among teachers are more important for female teachers than for male teachers (Kruse et al., 1994; Mora-Ruano et al., 2018). This includes encouraging and cooperating in community development, enjoying common work, exchanging expertise, and interest in collaboration with colleagues (Kruse et al., 1994; Mora-Ruano et al., 2018). When these initiatives cannot be met, it may lead to cynicism toward the professional community. However, no gender differences were found in terms of exhaustion, which contradicts the results of some previous studies (Antoniou et al., 2006). It has been suggested that gender role differences tend to be cultural rather than biological (Garcia-Arroyo et al., 2019), therefore, it may be useful to undertake further study of the cultural values of gender differences in various aspects of teacher burnout. Finally, the findings showed that teachers’ professional agency did not mediate the association between gender and burnout. This might be because of the small effect size of the relationship between teachers’ professional agency in the classroom and burnout, the mediator role of professional agency between gender and burnout can be explored further.

### 5.3 Practical implications

It has been revealed in this study that creating a collaborative learning environment by transforming teaching practices and active reflection in classroom seem to constitute Chinese early career teachers’ professional agency in the classroom. Thus, to promote teachers’ professional agency, it is important to emphasize the complexity of teachers’ professional agency. For example, teacher education programmes could provide teachers various opportunities to learn how to build a collaborative classroom environment and reflect on their teaching practices. The present study showed that early career teachers’ professional agency seems to be negatively related to their burnout, and therefore facilitating professional agency might function to reduce teacher burnout. Other preconditions for teachers’ burnout also need to be considered to support teachers’ well-being (Grayson and Alvarez, 2008).

## Data Availability

The original contributions presented in the study are included in the article/supplementary material, further inquiries can be directed to the corresponding author.
